# Effects of polypropylene micro- and nanoplastics from surgical masks in amphibian and echinoderm models

**DOI:** 10.1007/s10646-026-03077-w

**Published:** 2026-04-02

**Authors:** Renato Bacchetta, Clotilde Vacchelli, Arianna Pica, Francesco Saliu, Daniela Maggioni, Francesco Bonasoro, Nadia Santo, Paolo Tremolada, Michela Sugni

**Affiliations:** 1 Department of Environmental Science and Policy, Università degli Studi di Milano, Via Celoria, 26, Milan, 20133 Italy; 2 Department of Earth and Environmental Sciences DISAT, Università degli Studi di Milano Bicocca, Piazza della Scienza, 1, Milan, 20126 Italy; 3 Department of Chemistry, Università degli Studi di Milano, Via Golgi, 19, Milan, 20133 Italy; 4 Imaging Facility, Unitech NOLIMITS, Università degli Studi di Milano, Via Golgi, 19, Milan, 20133 Italy

**Keywords:** Face mask, Plastic pollution, Toxicity, Effects, Correlative microscopy, COVID-19 pandemic

## Abstract

Micro- and nanoplastic toxicity is a growing environmental concern, especially in aquatic ecosystems. This study investigates the acute and chronic effects of polypropylene microfibers (PP MFs), derived from artificially aged surgical face masks, on two aquatic species: the freshwater amphibian *Xenopus laevis* and the marine brittle star *Ophiactis virens*. Characterisation of the aged PP material revealed both microfibers (mean size: 21.7 ± 13.4 μm × 1.9 ± 0.8 μm) and nanofragments (mean diameter: 176 ± 28 nm), with a nano/micro abundance ratio of 4.3. *X. laevis* larvae ingested large quantities of PP MFs, resulting in near-complete gut obstruction, while juvenile *O. virens* did not show internal accumulation. Despite this, both species exhibited some sublethal effects, particularly at the behavioural level and at the highest tested dose (10 µg mL⁻¹). Indeed, *X. laevis* larvae reduced their swimming activity, whereas *O. virens* showed impaired righting ability. Moreover, *X. laevis* larvae grew significantly more than controls at all tested concentrations (0.1–10 µg mL⁻¹). No significant effect was observed at the cell-tissue level, except for a few cases of ultrastructural alteration in *X. laevis* intestinal epithelium. The LOEC values were 0.1 µg mL⁻¹ for *X. laevis* and 10 µg mL⁻¹ for *O. virens*; corresponding NOECs were < 0.1 and 1.0 µg mL⁻¹, respectively. These findings highlight species-specific responses, likely linked to ecological traits and life stage, and caution against generalizing toxicity outcomes across taxa. Overall, the study underscores the environmental legacy of COVID-19-related waste able to release a huge quantity of both micro- and nano-plastics with variable yet significant impacts on aquatic life.

**Supplementary Information:**

The online version contains supplementary material available at 10.1007/s10646-026-03077-w.

## Introduction

Plastic pollution has emerged as a critical environmental issue, posing a serious threat to biodiversity, ecosystems, and human health (Bocker and Silva 2025). Plastic debris now contaminates virtually every habitat, from marine and freshwater bodies to terrestrial environments, with estimates exceeding 170 trillion plastic particles floating in the oceans alone (Isa et al. 2023). A substantial portion of this pollution consists of microplastics (MPs; <5 mm) and nanoplastics (NPs; <1000 nm), which originate from both primary sources (intentionally produced) and secondary degradation of larger plastic items due to UV radiation and mechanical abrasion (Wang et al. 2021).

The COVID-19 pandemic has exacerbated this issue by introducing large volumes of personal protective equipment (PPE), particularly face masks. In early 2020, China alone produced over 116 million masks daily, dramatically increasing PPE waste (Qualhato et al. 2024). Surgical masks typically consist of a three-layer polypropylene (PP) structure—a hydrophobic outer layer, a filtering middle layer, and a soft inner layer—designed for effective protection but resistant to degradation and recycling (Das et al. 2021).

Once discarded, these masks undergo photodegradation, fragmenting into MPs and NPs that contaminate aquatic environments (Wang et al. 2021). High concentrations of mask-derived particles have been detected in freshwater systems, coral reefs, and coastal areas, sometimes exceeding 10,000 particles per cubic meter (Erni-Cassola et al. 2019).

Despite growing attention to microfiber impacts, limited research has focused specifically on plastics released from degraded face masks. Available studies reveal mixed effects: for example, exposure to mask-derived MPs caused immune and metabolic alterations in terrestrial invertebrates (Kwak and An 2021; Jemec Kokalj et al. 2022) and oxidative stress in aquatic microalgae (Ma et al. 2021; Das et al. 2023). Marine invertebrates and fish have been shown to ingest these particles, leading to intestinal accumulation, reduced reproduction, and behavioral changes (Sun et al. 2021; Isa et al. 2023). In zebrafish, exposure caused cardiotoxicity, changes in gene expression, and potential endocrine disruptions (Sendra et al. 2022; Qualhato et al. 2024).

In the context of the COVID-19 pandemic, Qualhato et al. (2023) emphasized the need to expand nanoplastics research to less-studied taxa such as amphibians. In response, our study investigates the ecotoxicological effects of PP-derived micro- and nanoplastics on two contrasting aquatic models: *Xenopus laevis* (a freshwater amphibian widely used in toxicology) and *Ophiactis virens* (a brittle star increasingly recognized as a sentinel marine invertebrate). The use of environmentally relevant samples, two different ecotoxicological models and several endpoints (behavioural and histological) allows for inquiry, into the complexity of the micro- and nanoplastic toxicity, in comparable way. Despite the need of standardization (necessary for regulation), the complexity of the issue and the heterogeneity of the material still need broad-spectrum research.

*X. laevis* is a well-established model for evaluating contaminant toxicity, including plastics like polystyrene, polyester, and PET (De Felice et al. 2018; Bacchetta et al. 2021; Cai et al. 2024). *O. virens*, a benthic echinoderm with deuterostome affinities, features traits advantageous for toxicological studies: developmental plasticity, regenerative capacity, and ecological significance in marine benthic communities (Sugni et al. 2008; Rakaj et al. 2021; Rosner et al. 2024).

By studying the response of vertebrate and invertebrate species from distinct aquatic environments, this study aims to evaluate the species-specific effects of environmentally aged PP MFs, providing an integrative assessment of plastic particle toxicity relevant to post-pandemic pollution scenarios.

## Materials and methods

### Sample preparation and characterization

Polypropylene samples were prepared following the protocol of Saliu et al. (2021). Inner PP layers from surgical masks were cut into small fragments and subjected to artificial ageing under UV-A lamps (340 nm, 0.76 W/m²/nm) at 65 °C for 180 h. This treatment simulated environmental weathering and resulted in a mixture of micro- and nanoplastics.

Dynamic Light Scattering (DLS) was used to characterise the behaviour and aggregation of particles in two media: FETAX solution (for *X. laevis*) and filtered artificial seawater (FASW, for *O. virens*). FETAX composition in µg mL^-1^ was: 625 NaCl, 96 NaHCO₃, 30 KCl, 15 CaCl₂, 60 CaSO₄·2 H₂O, and 70 MgSO₄. FASW was prepared by dissolving in deionized water the commercial Instant Ocean salt mixture, until reaching a salinity of 37‰. DLS measurements were taken at 0, 6, 24, and 48 h post-preparation using a Zetasizer Nano ZS. Particle size and distribution were assessed assuming the solvent’s refractive index (1.33) and viscosity (0.89), and a solute refractive index of 2.42.

Morphological analysis was conducted using scanning electron microscopy (SEM, FE-SEM Sigma, Zeiss). Fibers were sputter-coated with platinum and imaged at 1000x magnification for microplastics and 6500x–40,000x for nanoplastics. ImageJ software (Schneider et al. 2012) was used to measure fiber dimensions. Nanoparticle size was derived from particle area, assuming spherical shape. The validity of the spherical approximation was assessed by comparing measured and calculated particle perimeters, the latter geometrically obtained from the assumed spherical shape. The ratio of nanoparticles to microfibers was estimated across 20 SEM fields.

The number of fibers per mL was estimated using the nominal concentration and the average mass of a single fiber, calculated from its cylindrical volume and polypropylene density (0.91 g/cm³). The volume of each fiber was geometrically derived, assuming a cylindrical shape, from the length of each fiber and its width (assumed as diameter). The mean mass per fiber (µg fiber^-1^) was calculated considering the relative abundance of each dimensional class and the mean microfiber weight within each class.The mass contribution of nanoparticles was considered negligible.

### Preparation of exposure suspensions

Exposure concentrations were set at 0.1, 1.0, and 10 µg mL^− 1^. The lowest concentration reflected environmentally relevant levels (Naidoo and Glassom 2019), while those of 1.0 and 10 µg mL^− 1^ were selected to represent possible conditions of pollution hotpots.

Stock solutions (10 µg mL^-1^) were prepared in FETAX solution (for *X. laevis*) and FASW (for *O. virens*) and diluted to final concentrations. A probe sonicator was used to disperse aggregates. Solutions were stored at 4 °C and re-sonicated before use.

### Animal maintenance and exposure

Adult *X. laevis* were maintained in a controlled system at the University of Milan (T = 20 ± 2 °C; pH 7.5 ± 0.5; conductivity = 1000 ± 100 µS/cm; 12 h light/dark). Embryos were obtained through natural mating and selected at Nieuwkoop and Faber, NF, stage 8–9 (Nieuwkoop and Faber 1994). Ten embryos per Petri dish (60 mm, 10 mL) were exposed to control or test suspensions with five replicates per treatment (*n* = 50 embryos/treatment). The exposure lasted until NF stage 46 (~ 100 hpf), with daily media renewal. Mortality, malformation, and growth inhibition were recorded.

Juvenile *O. virens* were collected from the Gulf of Le Grazie (Ligurian Sea, Italy) and acclimated in 50-L aquaria for three weeks at 17 °C, the average annual surface temperature of the Mediterranean Sea. Individuals (~ 2 mm disc diameter) without recent fission or regeneration were selected and anaesthetised in a 3.5% MgCl_2_ solution (in 1:1 FASW and distilled water). Two adjacent arms per animal were amputated at the junction between the third and fourth segment from the central disc. Amputated animals were transferred to glass container filled with FASW to recover from anaesthesia and subsequently randomly assigned to treatments (*n* = 20 per treatment, two replicates). All glass containers were provided with oxygenation stones and mussel shells as shelter. Exposure lasted 14 days, with 48-hour media changes. Animals were fed spirulina thrice weekly; mortality was recorded daily.

### Behavioural testing

At the end of exposure, behavioural assessments were conducted for both species. *X. laevis* larvae were filmed for 60 s after a 20-second acclimation period. Five individuals per replicate (*n* = 25 per treatment, 100 total) were recorded using a 1080p HD camera. Videos were analyzed with the Animal Tracker plugin in ImageJ (Gulyás et al. 2016) to evaluate total immobility time, distance moved, and average swimming speed in inner and outer zones of the test arena.

For *O. virens*, righting ability and locomotion were assessed. Righting time was defined as the time needed to reorient from an inverted position (oral side up) to the correct posture. Locomotion was recorded during a 3-minute trial and quantified as horizontal displacement speed. Both endpoints were video-recorded and analyzed using the same analytical software as for *X. laevis*.

### Developmental assessments

In *X. laevis*, surviving larvae were anaesthetised with MS222 and then photographed under a Leica EZ4 D stereomicroscope. Total length (head to tail) was measured using FETAX test guidelines (ASTM, 1998) and statistically analysed.

In *O. virens*, arm regeneration was used as a proxy for developmental toxicity. Measurements of regenerating arms from samples anaesthetized with 3.5% MgCl_2_ included regenerated arm length (L), stump diameter (d), and the number of regenerated segments (Ns). Regeneration efficiency was expressed through a differentiation index (Di), calculated as:

Di = Ns/Ln.

where Ln represents the normalised length of the regenerate, calculated using the ratio between the length (L) and the diameter of the arm stump (d), to eliminate the effects of age or size, as follows:

Ln = L/d.

### Histological analysis

Ten *X. laevis* larvae per group (*n* = 40 total) and eight *O. virens* per group (*n* = 32 total) were fixed in Bouin’s fluid (overnight at room temperature for *X. laevis*, 3 weeks at 4 °C for *O. virens*). After dehydration and paraffin embedding, 7 μm transverse sections were cut and stained: Hematoxylin-Eosin for *X. laevis* and Milligan’s trichrome for *O. virens*. Serial sections of *X. laevis* were taken from three different portions of the larvae, focusing on different organs: (1) head, including eyes and gills; (2) otic vesicles and heart; (3) visceral area (Fig. S1). Sections of *O. virens* were mainly focused on the central disc, which contains the digestive system. All slides were observed using a Leica-equipped light microscope.

### Scanning electron microscopy (SEM)

Twenty *X. laevis* larvae and eight *O. virens* per group were fixed using 2.5% glutaraldehyde and 4% paraformaldehyde, then post-fixed in 1% OsO₄. After dehydration and critical point drying, tissues (gut and gills in *X. laevis*; central disc and regenerating arms in *O. virens*) were mounted onto standard aluminium stubs and coated with a 5 nm platinum layer for SEM imaging (FE-SEM Sigma, Zeiss).

A correlative microscopy analysis was also performed to study in more detail the interactions between PP fibers and the digestive epithelia of the exposed samples. A selection of slides from the histological study was further analysed by SEM. These were placed in xylene for about one week until the coverslips detached. After a brief rinse in clean xylene, slides were air-dried and stored in the dark until analysis.

### Statistical analysis

In *X. laevis*, mortality and malformation rates were compared using Fisher’s exact test. Larval length was analyzed via General Linear Model (GLM), with treatment as a fixed factor and replicate as random. Behavioral endpoints were assessed using Kruskal-Wallis and Mann-Whitney tests.

In *O. virens*, mortality was evaluated using chi-square test with Yates’ correction. Logistic models were applied to evaluate the effects of concentrations and replicates on the mortality rate. One-way ANOVA was used for behavioral and regenerative endpoints (righting time, speed, differentiation index), followed by Tukey’s post-hoc test. Data normality was checked using the Shapiro-Wilk test.

Significance was accepted at *p* < 0.05. Analyses were conducted using SPSS 15.0 and RStudio.

## Results

### Characterization of the PP microfibre sample

**Fig. 1:**
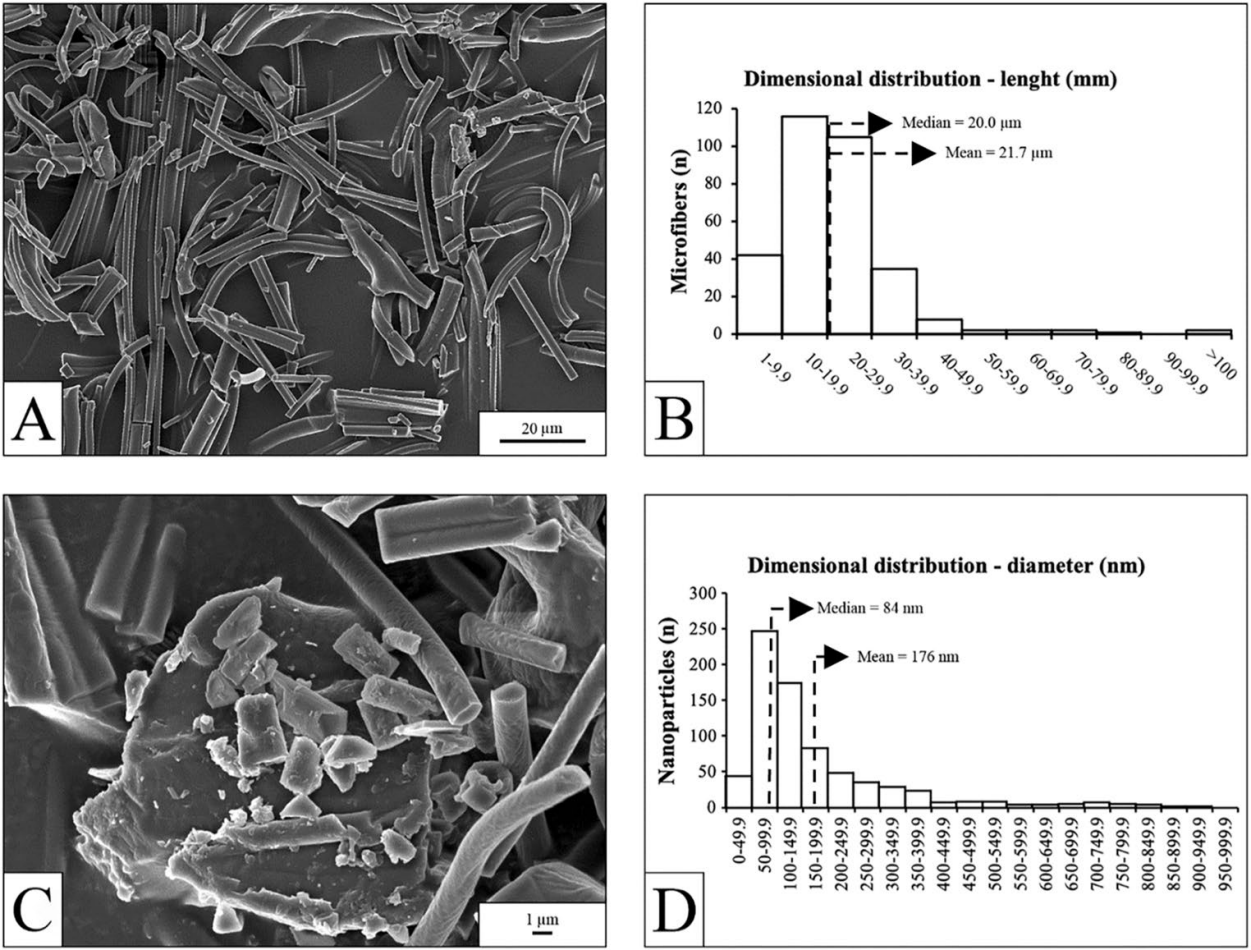
Characterization of the PP microfiber sample used for the exposure tests. (**A**), SEM image of the sample at low magnification. (**B**), size distribution of microfiber lengths greater than 1 mm. (**C**), SEM image at higher magnification. (**D**), size distribution of the nanometric fraction, expressed as the diameter of equivalent spherical nanoparticles

In the SEM images (Fig. 1A), PP microfibers exhibited a cylindrical shape with a mostly uniform diameter, resulting from the fragmentation of a highly homogeneous PP filament. Fiber length ranged from 3.5 μm to 113.3 μm, with an average of 21.7 ± 13.4 μm (SD) and a median of 20.0 μm (Fig. 1B); approximately 95% were shorter than 40 μm. Widths ranged from 0.7 to 6.7 μm (mean 1.9 ± 0.8 μm; median 1.7 μm, Fig. S2). At higher magnification (Fig. 1C), small fragments were observed: larger ones retained a cylindrical shape, while smaller ones appeared irregular and fell within the nanometric range, likely originating from multilayer fragmentation or secondary breakdown of smaller particles. According to Saliu et al. (2021), secondary degradation was the major pathway for nanoplastic generation. This pathway is consistent with evidence from studies on chemical and mechanical weathering (Qualhato et al. 2023; Isa et al. 2023; Hu et al. 2024). Due to their irregular shape, the nanoparticle size was characterised using the “equivalent diameter,” geometrically calculated from the particle area assuming a spherical shape. Among 743 particles analysed, only three were in the micrometric range (up to 1.45 μm), while 740 ranged from 23 to 968 nm. The average nanoparticle size was 176 ± 28 nm (SD), with a median of 84 nm; approximately 74% measured < 200 nm (Fig. 1D). The broad size distribution, highlighted in Fig. 1, closely traced the heterogeneity of environmental samples. Additionally, the origin of the sample—UV-driven degradation of surgical masks—further supports this proximity and, thus, the ecological relevance of this sample for ecotoxicological studies, as highlighted by Bocker and Silva (2025).

To assess the nanoparticle-to-microfiber ratio, counts were performed in the same high-resolution SEM fields used for size analysis. The median and mean nano/micro ratios were 4.3 and 20.9, respectively; the higher value of the mean resulted from one outlier image with an exceptionally high nano/micro ratio. Thus, to avoid overestimation, the median was used to estimate the particle count per mL in the exposure media at each concentration. For the three exposure concentrations of 0.1, 1.0, and 10 µg mL^-1^, the total number of particles in the exposure media were respectively: 7,330 (1,380 micro and 5,950 nano items), 73,300 (13,800 micro and 59,500 nano items) and 733,000 (138,000 micro and 595,000 nano items). Despite that nanoparticles were 4.3 times more numerous than microfibers, they contributed less to the total mass. Infact, calculating their mass, the results were largely negligible. These findings highlight the importance of evaluating both particle number, which is enphasised by smaller particles, and mass (largely dependent by larger particles) in ecotoxicological assessments, to provide a complete picture of the exposure experiment.

**Fig. 2:**
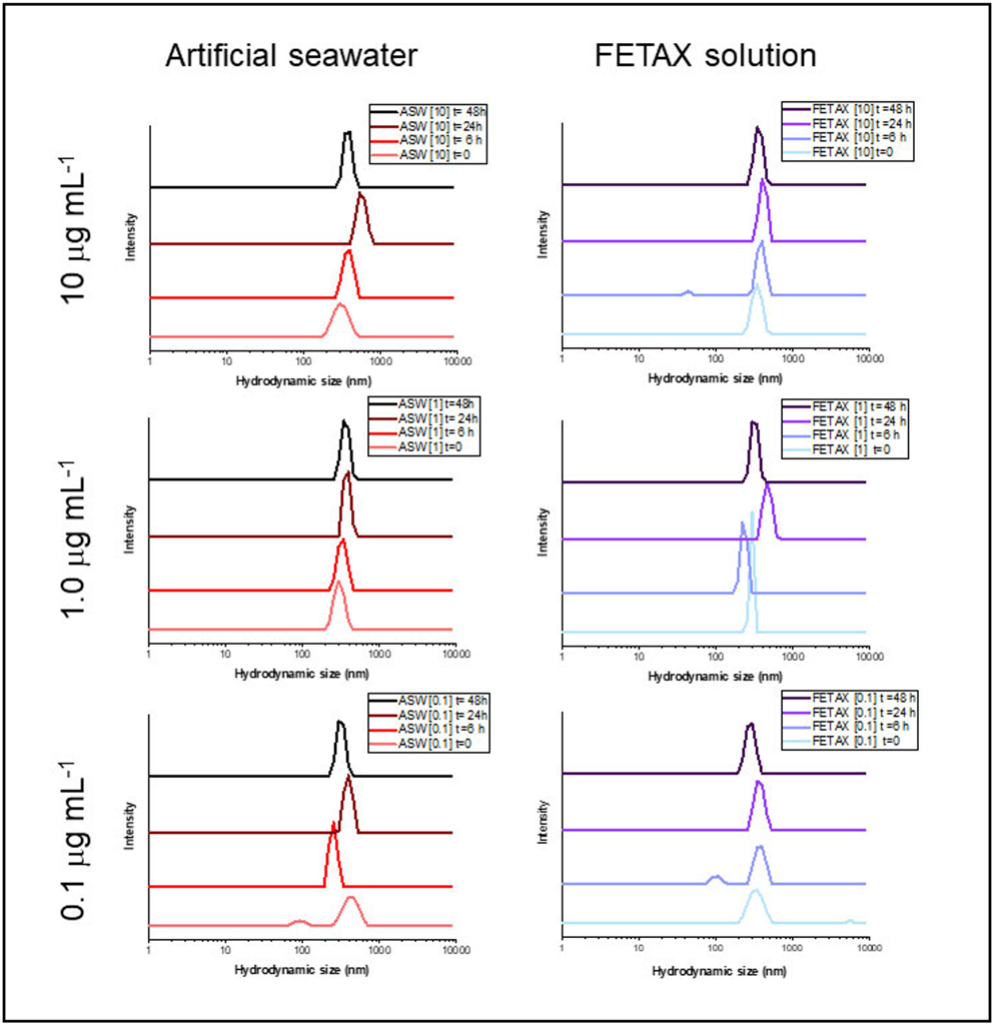
Dynamic Light Scattering (DLS) analysis of the PP microfiber sample in artificial seawater (ASW, used for *O. virens*) and FETAX solution (used for *X. laevis*) at three concentrations (0.1, 1.0, and 10 µg mL^-1^) over four different time points (0, 6, 24, and 48 h)

DLS analysis (Fig. 2) revealed a consistent hydrodynamic diameter peak of ~ 300–400 nm across media (FETAX and ASW) and time points (0–48 h), with reduced peak intensity at lower concentrations. The larger hydrodynamic diameter compared to the median nanoparticle size (84 nm), observed in both media (FETAX and FASW), suggests aggregation phenomena in both suspensions and a similar behaviour of the material (PP microfiber sample) in the two media. Similar behaviours have been reported in marine and freshwater media (Qualhato et al. 2023), where ionic strength, organic matter, and biological interactions influence aggregation dynamics.

### Exposure experiments

Both model species, *X. laevis* and *O. virens*, were exposed to PP microfibers at 0.1, 1.0, and 10 µg mL⁻¹. No significant differences in mortality or malformation rates were found in *X. laevis* exposed specimens in comparison to controls (Fisher’s exact test, *P* > 0.24; Table S1). In *O. virens*, mortality was also unrelated to treatment (χ² = 2.46, df = 3, *P* = 0.48; Table S2) and logistic regression confirmed no significant effect between control and exposure samples (*P* = 0.30), nor differences among replicates (*P* = 0.22). Control mortality remained within acceptable limits.

### Development parameters

**Fig. 3:**
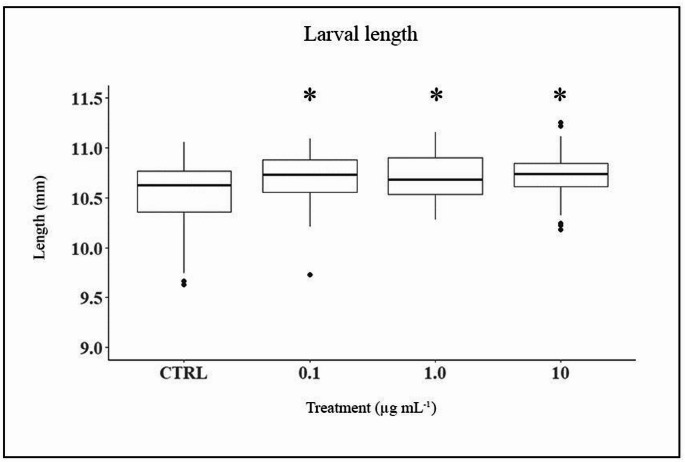
Box plot showing the body length of *X. laevis* larvae across the different treatments. Asterisks indicate significant difference from control

In *X. laevis*, larval length (*n* = 197; Table S3) was analyzed using a General Linear Model (GLM). No significant effects were detected for replicates (F₄,₁₉₅ = 0.33, *P* = 0.85) or replicate-treatment interaction (F₄,₁₉₅ = 1.1, *P* = 0.34), confirming data consistency. However, PP exposure significantly affected larval length (F₃,₁₉₆ = 4.3, *P* = 0.028). Tukey’s post-hoc test indicated that larvae in all exposure groups were significantly longer than controls (Fig. 3), although no differences were found among concentrations. Marginal means were: control = 10.55 mm (95% CI: 10.47–10.62), 0.1 µg mL⁻¹ = 10.70 mm (95% CI: 10.62–10.77), 1.0 µg mL⁻¹ = 10.73 mm (95% CI: 10.65–10.80), 10 µg mL⁻¹ = 10.72 mm (95% CI: 10.65–10.79).

**Fig. 4:**
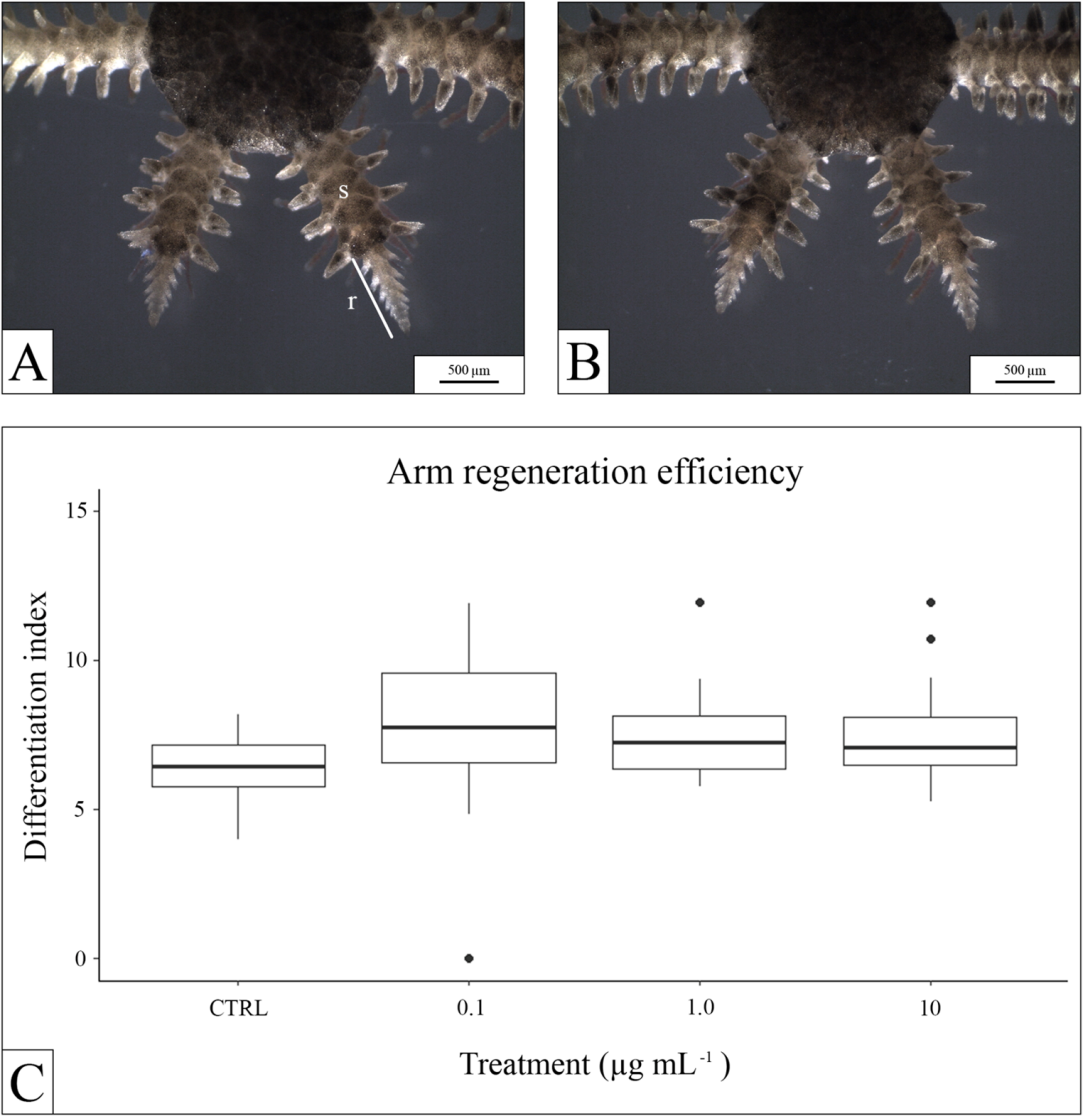
Stereomicroscopic images of regenerated arm tips: (**A**), control sample (**B**), sample exposed to 10 µg mL^-1^ PP. (**C**), box-plot showing the length of the regenerated arms across the different treatments. s = stump; r = regenerate

In *O. virens*, arm regeneration was assessed using the differentiation index (regenerated segments normalized to length). Regeneration appeared slightly reduced in exposed groups, but differences were not significant (ANOVA F₃,₁₁₂ = 2.49, *P* = 0.064; Fig. 4).

### Behavioural parameters

In *X. laevis*, video tracking provided several behavioural parameters, such as: *i*) time spent in the inner and outer parts of the arena; *ii*) time spent moving (swimming time); *iii*) total distance travelled, and *iv*) the mean swimming speed (Table S4). No significant difference in arena zone preference was observed (χ² = 4.4, df = 3, *P* = 0.22). Most larvae stayed most of the time (> 50 s out of 60) in the outer arena zone. Swimming time was reduced in exposed larvae, particularly at 10 µg mL⁻¹ (median: 14.4 s) compared to controls (23.6 s; Mann-Whitney z = − 2.14, *P* = 0.032). Lower exposure groups showed similar trends, but without statistical significance. Total distance travelled was also reduced: medians were 478 mm (control), 167, 180, and 67 mm for 0.1, 1.0, and 10 µg mL⁻¹ respectively. The 10 µg mL⁻¹ group differed significantly from controls (z = − 2.31, *P* = 0.021). No significant differences were found in mean swimming speed (χ² = 1.8, df = 3, *P* = 0.61).

**Fig. 5:**
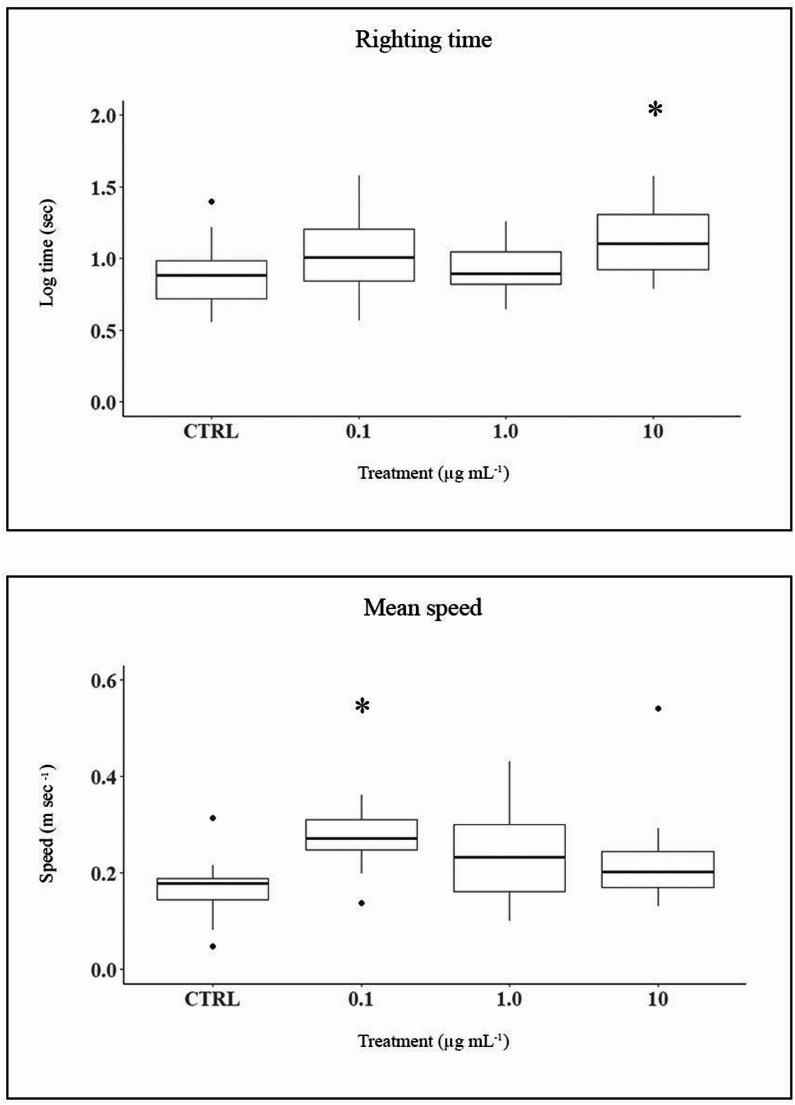
Box plots showing log-transformed righting time (upper panel) and mean locomotion speed (lower panel) of *O. virens* individuals across treatments. Asterisks indicate significant difference from control (*p* < 0.05)

In *O. virens*, righting time (Table S5) increased significantly at 10 µg mL⁻¹ (GLM F₃,₅₅ = 3.15, *P* = 0.032; Tukey *P* = 0.027; Fig. 5A). Data normalization was confirmed by Kolmogorov–Smirnov test. No significant effects were found for replicates or interactions. Locomotion speed also differed among groups (ANOVA F₃,₅₉ = 4.58, *P* = 0.006), with significantly higher speed at 0.1 µg mL⁻¹ vs. control. Other pairwise comparisons were not significant (Fig. 5B).

### Histological analysis

**Fig. 6:**
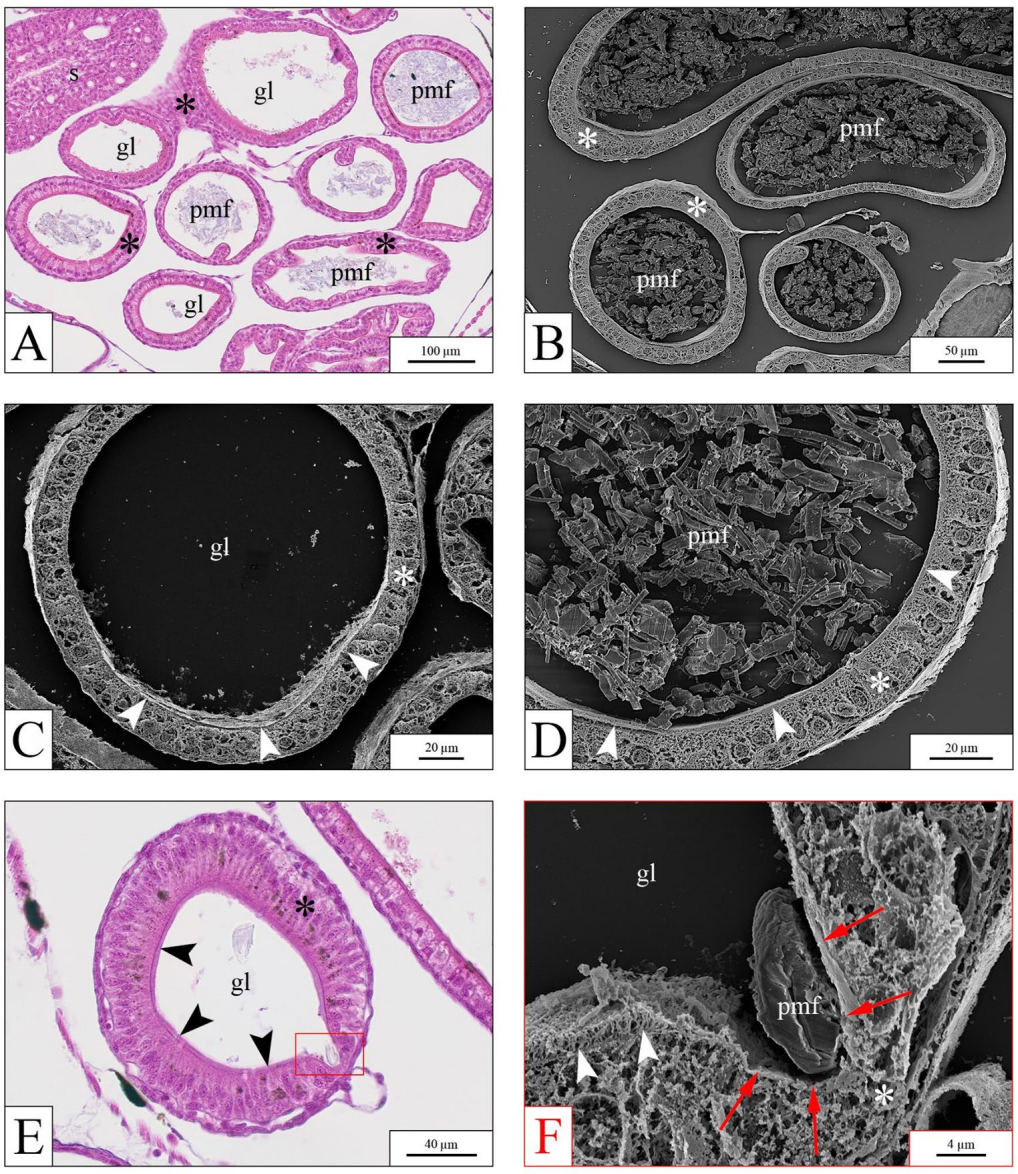
Histological and ultrastructural analysis of the visceral region in *X. laevis* larvae following PP exposure. (**A**), light microscopy image of a histological section from a larva exposed to 10 µg mL⁻¹ PP, showing extensive accumulation of microfibers in the intestinal loops. (**B**), SEM image of the intestinal loops from a larva exposed to 10 µg mL⁻¹ PP. (**C**), SEM image of an intestinal loop from an unexposed control larva, showing an empty gut lumen. (**D**), SEM image at intermediate magnification of an intestinal loop from a larva exposed to 10 µg mL⁻¹ PP, displaying a dense aggregation of microfibers within the lumen. (**E**–**F**), correlative analysis of an intestinal loop from a larva exposed to 0.1 µg mL⁻¹ PP, showing a single microfiber pressing against and disrupting the intestinal wall structure. (**E**), transverse section of the small intestine showing a microfiber embedded in the digestive epithelium. (**F**), higher-magnification SEM image of the tissue area indicated in panel E (red box), revealing disruption of the brush border in epithelial cells in direct contact with the microfiber (red arrows). gl = gut lumen; pmf = polypropylene microfiber(s); s = stomach; * = intestinal wall; arrowheads = brush border

In *X. laevis*, histological analysis covered three body regions: the head level, including the eyes and gills (Fig. S1-1); the region containing the otic vesicles and heart (Fig. S1-2); and the visceral area, including stomach, liver, pancreas, and the intestinal loops (Fig. S1-3). The gut appeared to be the primary target organ following exposure to PP microfibers (*n* = 5/10 at 0.1 µg mL⁻¹; *n* = 4/10 at 1 µg mL⁻¹; and *n* = 9/10 at 10 µg mL⁻¹; Fig. 6). Both the large and small intestine, defined according to Chalmers and Slack (1998), showed significant microfiber accumulation (Fig. 6A and B). The presence of PP microfibers became more pronounced with increasing exposure concentration, with the intestinal loops of larvae exposed to the highest PP concentration being filled with microfibers (Fig. 6D), unlike from the controls, in which they appeared empty (Fig. 6C). Occasionally (*n* = 4/30), fibers contacted the intestinal wall, compressing the brush border and distorting the cell architecture (Fig. 6E). These samples were further analysed by correlative (SEM) analyses to verify any actual evidence of cell damage (see below). Gills showed no fiber accumulation.

**Fig. 7:**
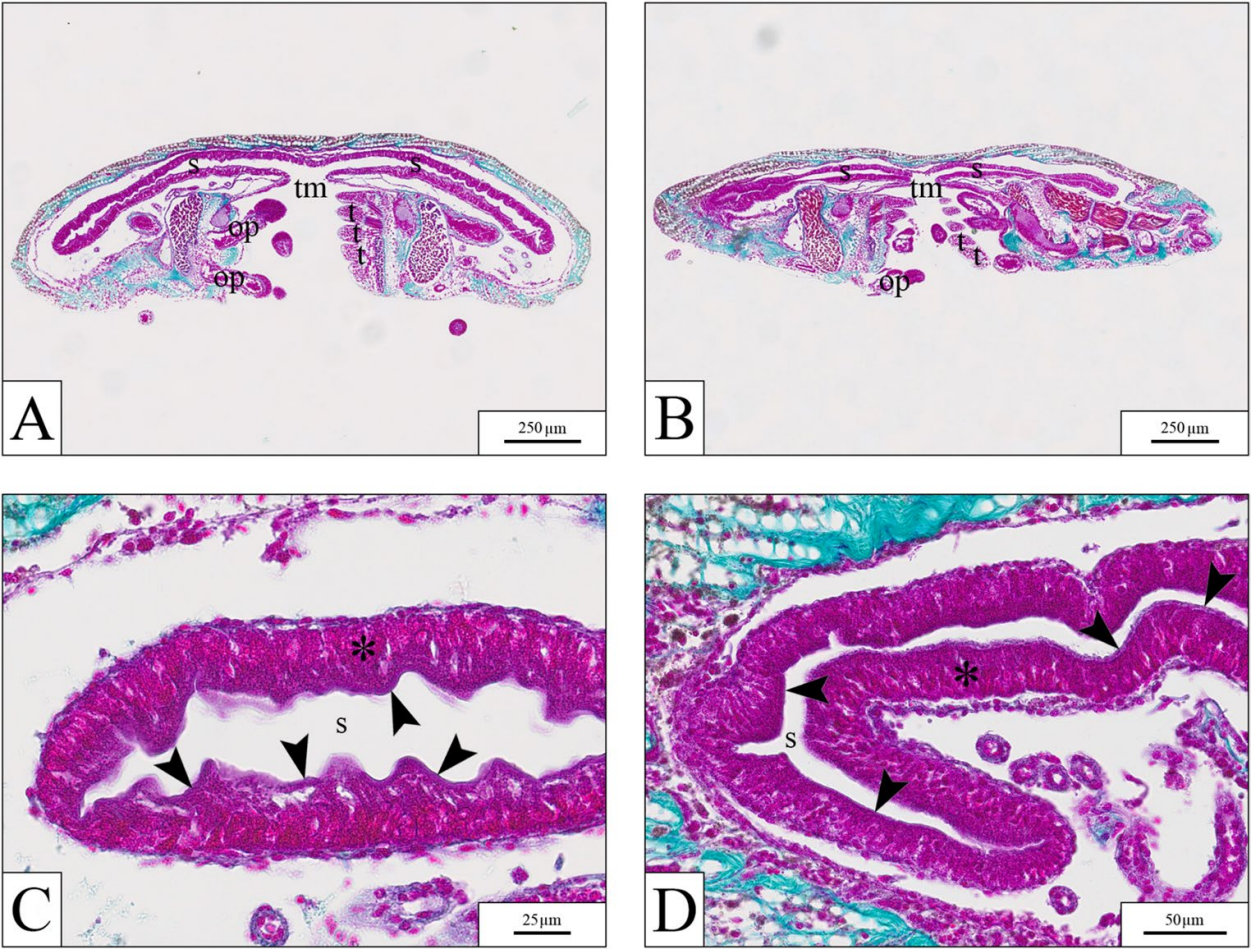
Histological sections of *O. virens* samples at low (**A**–**B**) and high magnification (**C**–**D**). No histological alterations were observed between control (**A**, **C**) and specimens exposed to 10 µg mL⁻¹ PP (**B**, **D**). op = oral podia; s = stomach; t = teeth; tm = true mouth; * = stomach epithelium; arrowhead = brush border

In *O. virens*, no histological differences were observed between controls and exposed groups, even at 10 µg mL⁻¹ (Fig. 7). Stomach epithelium remained intact in all cases.

### Scanning electron microscope (SEM) – correlative analyses

In line with the histological observations, SEM analyses of selected *X. laevis* slides revealed intestinal loops filled with PP microfibers, in most cases without apparent damage to the intestinal wall (Fig. 6B and D). In those samples (4/30) where histological analyses showed the distortion of the intestinal epithelium due to fibers compression (see above), correlative analysis could confirm actual damage to the apical portion of the cells (destruction of the brush border; Fig. 6E and F). In other samples no ultrastructural alterations were observed despite the direct contact between cells and fibers.

**Fig. 8:**
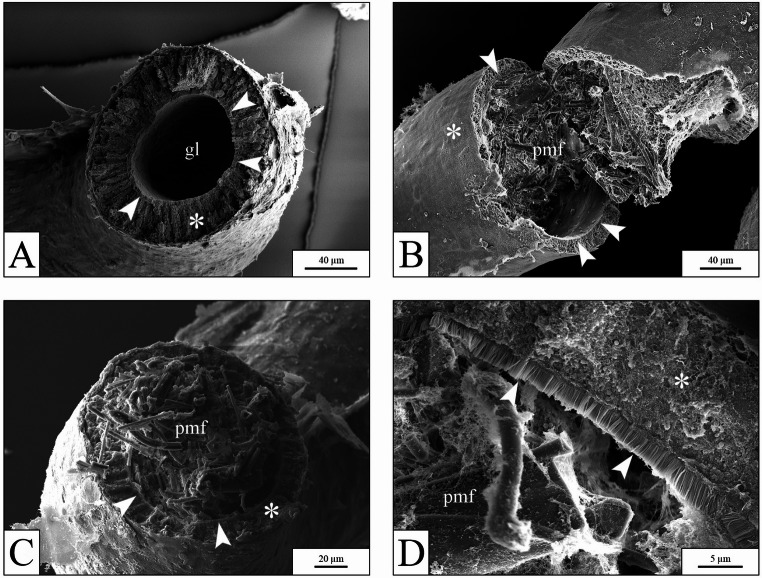
SEM images of intestine from *X. laevis* larvae. (**A**), empty intestinal lumen in a control larva. (**B**-**C**), intestines of larvae exposed to 10 µg mL⁻¹ PP showing dense microfiber accumulation. (**D**), detail of the gut in a larva exposed to 10 µg mL⁻¹ PP, showing the intact structure of the brush border and the presence of mucus among fibers. gl = gut lumen; * = intestinal wall; arrowheads = brush border; pmf = polypropylene microfibers

SEM analysis of the *X. laevis* gut confirmed substantial accumulation of microfibers in exposed samples (Fig. 8).

While the intestines of control larvae were empty (Fig. 8A), the digestive tracts of larvae exposed to the highest PP concentration (10 µg mL⁻¹) were completely obstructed by ingested microfibers (Fig. 8B and C). However, no malformations or structural damage were observed at the level of the gut epithelium, even under higher magnification. The microvilli layer appeared intact, and the microfibers were entirely covered by intestinal mucus (Fig. 8D). Analysis of the *X. laevis* gills revealed no accumulation of microfibers or damage to the gill structure. Only occasionally, individual microfibers were detected within the gill apparatus (Fig.S3 ).

**Fig. 9:**
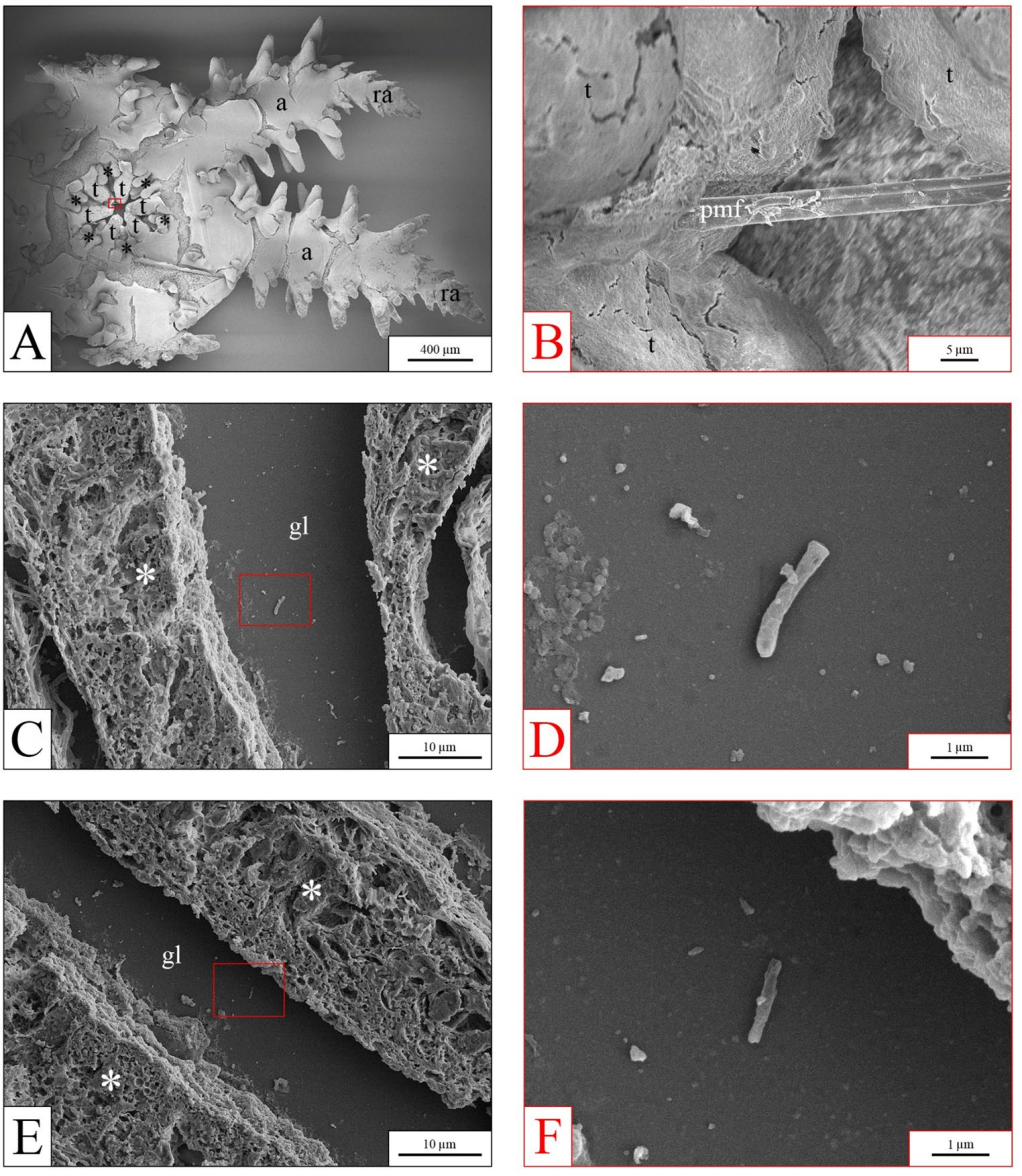
SEM images from *O. virens* samples. (**A**-**B**), low and high magnification images of the oral region from a sample exposed to 10 µg mL⁻¹ PP. (**A**), oral view. (**B**), detail of the area indicated in panel A (red box), showing PP microfibers among the teeth. (**C**-**D** and **E**-**F**): low (**C**-**E**) and high magnification (**D**-**F**) SEM images of the stomach in histological slides from samples exposed to 10 µg mL⁻¹ PP, revealing isolated fragments of PP microfiber in the stomach lumen. black asterisk = oral podia; t = teeth; a = arm; ra = regenerated arm; pmf = polypropylene microfibers; white asterisk = stomach wall; gl = stomach lumen

In the *O. virens* experiment, PP microfibers were detected only at the highest concentration (10 µg mL⁻¹), specifically in the pre-oral opening and between the teeth (Fig. 9A and B). No microfibers were observed at lower concentrations. In some slides prepared for SEM analysis, small fragments of PP microfibers were found within the stomach lumen of specimens exposed only to the highest PP concentration (Fig. 9C–F); however, no accumulation was ever observed.

## Discussion

Microplastic ingestion is reported to cause multiple adverse effects, such as metabolic disruption, changes in feeding behavior, and developmental or reproductive impairments, depending on the species (Bom and Sá 2021; Issac and Kandasubramanian 2021; Hossain and Olden 2022). *X. laevis* larvae showed no increase in mortality or malformations. Likewise, *O. virens* exhibited no mortality or impairment in arm regeneration, although a minor reduction in regrowth was observed at the highest concentration. These results are consistent with prior studies reporting limited acute toxicity from MPs at environmentally relevant concentrations (De Felice et al. 2018; Sugni et al. 2024; Bacchetta et al. 2024). Nonetheless, both models displayed subtle behavioural and physiological effects.

In *X. laevis*, larvae exposed to PP fibers were significantly longer than controls. Several hypotheses could account for this observation: (i) microbial colonization on fibers providing an auxiliary food source in exposed specimens in respect to control (no fiber addition); (ii) induced satiety due to gut filling reducing the need of feeding and, thus, the energy expenditure for searching food sources; (iii) the presence of endocrine-active substances leaching from PP, promoting growth in exposed specimens in respect to control (Qualhato et al. 2023; Lin et al. 2023b). Supporting hypothesis (ii), control larvae exhibited higher locomotor activity, possibly resulting in greater energy use and reduced growth. In contrast, PP-exposed larvae travelled shorter distances but at comparable swimming speeds—suggesting reduced activity rather than impaired motility, which does not support hypothesis (iii). This aligns with literature describing neuromodulatory effects of MPs without overt locomotor deficits (Qualhato et al. 2023). To elucidate this unexpected growth stimulation, future studies could analyze gut microbiota (16 S rRNA) to evaluate potential nutritional subsidies from fiber-colonizing microbes, while concurrently measuring energy reserves (e.g., lipid content) to assess growth-activity trade-off.

Divergent locomotor responses have been reported in zebrafish depending on MP source. While N-95 mask-derived MFs increased swimming velocity, surgical mask-derived MFs did not (Qualhato et al. 2024). In our study, total distance travelled decreased, but mean speed remained unchanged. This variability in behavioural outcomes reflects differences in species, exposure time, particle size, and MP type. For example, zebrafish larvae exhibited hypoactivity after exposure to 50 nm polystyrene NPs (Lin et al. 2023a), and reduced swimming in *Sebastes schlegelii* exposed to polystyrene beads was linked to gut accumulation and energy trade-offs (Yin et al. 2018).

In *O. virens*, a significant increase in righting time was observed at 10 µg mL⁻¹. This behavioural endpoint, widely used as a biomarker in echinoderms (Lawrence and Cowell 1996; Ardor Bellucci and Smith 2019), reflects neuromuscular coordination and organismal stress. The absence of physical accumulation of PP microfibers in *O. virens* specimens suggests that systemic or neurophysiological stress may be at play.

*Xenopus laevis* larvae showed a huge quantity of microfibers accumulated in their digestive tract (Figs. 6 and 8), confirming ingestion as one of the primary entrance routes of microplastics. Conversely, *Ophiactis vire*ns did not exhibit significant fiber accumulation, even at the highest concentration. Fibers observed in the pre-oral cavity, between teeth, and in the stomach lumen (Fig. 9) likely resulted from passive contact and incidental ingestion. The absence of microfiber accumulation in the gastrointestinal trac of *Ophiactis virens* excludes the active ingestion of these particles by this specis (or a selective rejection), as if they were able to recognize these particles as non-food items. This behaviour was already documented in marine invertebrates (Isa et al. 2023). Such interspecific differences, observed between *Xenopus laevis* larvae and *Ophiactis virens* specimens may reflect developmental stage or species-specific feeding strategies. Juvenile brittle stars may exhibit more selective feeding as they were already accustomed to feeding on other food sources. On the contrary, amphibian larvae have high nutritional demands (need to feed), and no prior feeding experience, because they start to feed during exposure. Moreover, they have an opportunistic feeding behaviour, ingesting a variety of edible particles (Altig et al. 2007). For example, they can filter algae and suspended particles from the water column, as well as scraping periphyton off the substrate (Altig et al. 2007). In addition, they are able to break ingested particles into suitable size fragments by their jaw sheaths and labial teeth (Wells 2007). The huge quantity of microfiber ingested in the few days of feeding at the end of the exposure period, confirmed their opportunistic feeding habits and wide- size range of ingested particles.

These patterns are consistent with (Balestrieri et al. 2022), who reported species-specific toxicity in amphibian larvae exposed to identical MP mixtures. While both species ingested MPs, only one showed reduced growth, hypoactivity, and increased mortality—even at 1.0 mg L⁻¹. Similarly, *X. laevis* larvae ingested PP microfibers proportionally to concentration, whereas *O. virens* did not. Despite high accumulation at 10 µg mL⁻¹, most fibers in *X. laevis* remained in the gut lumen. Mechanical compression of the brush border was observed, but no histopathological alterations occurred, in line with previous studies on polystyrene microplastics (De Felice et al. 2018) and PP fibers in zebrafish (Qualhato et al. 2024). SEM confirmed these findings: digestive cells appeared intact, even at points of contact with fibers. Only in one larva ( 0.1 µg mL⁻¹) structural deformations were observed, including loss of microvilli (Fig. 6E-F ). Fiber distribution was heterogeneous, with some intestinal loops blocked and others fiber-free. This observation suggests that the gastrointestinal anatomy may influence localized fiber retention but also that the high levels of clogging can delay or even prevent the transfer of the ingested material along the gastrointestinal tract until the anus. The long-term outcome of microfiber accumulation in *Xenopus laevis* gut can be only hypothesized: we can speculate that, in the case of severe clogging, tadpoles could even die because of starving. On a longer period of time, Balestrieri et al. (2022) observed high tadpole mortality after polyester microfiber exposure, concomitantly to high clogging events (high exposure concentrations). On the contrary, in the case of light clogging we can speculate that tadpoles could be able to eject indigested materials from the anus as non-digested residues. This has already been reported in several aquatic organisms, such as bivalves and fish (Choi et al. 2022; Dawson et al. 2025), in which high egestion rates were observed. Additionally, the latter authors found that in juvenile barramundi (*Lates calcarifer*), no translocation from the gastrointestinal tract occurred, indicating that MPs of different shapes and sizes are unlikely to accumulate in this species. In contrast, Choi et al. (2022) reported different results for the Pacific oyster (*Crassostrea gigas*), in which particles smaller than 50 μm showed the highest ingestion and egestion rates, allowing efficient depuration after 24 h, although not complete elimination of the particles. According to previous studies (Browne et al. 2008; Von Moos et al. 2012; Birnstiel et al. 2019), MPs accumulated in bivalves cannot be entirely discharged, and even prolonged egestion periods are insufficient to fully decontaminate bivalves following MP exposure (Ribeiro et al. 2017). Moreover, it must be considered that fiber morphology plays a key role in toxicity. Fibers tend to accumulate more readily than fragments or beads (Qiao et al. 2019). In *X. laevis*, physical damage caused by PVC fragments was linked to their rigidity and sharp edges (Bacchetta et al. 2024). In contrast, mask-derived PP fibers appeared softer and more flexible, causing intestinal clogging but no perforation. Previous work shown that polyester fibers from dryers can induce intestinal and body wall damage (Bacchetta et al. 2021), reinforcing the importance of fiber rigidity in determining toxicity outcomes. In conclusion, the degree of clogging may depend on several factors: such as dose, material features and shape (Gray and Weinstein 2017; Dawson et al. 2025; Kim et al. 2025).

SEM revealed a mucus coating on the ingested PP fibers, supporting previous studies reporting MP-induced gut inflammation and increased mucus secretion (Qiao et al. 2019; Hu et al. 2020; Li et al. 2020). This response, also observed in corals exposed to PP nanofibers (Isa et al. 2023), serves as a protective mechanism that reduces abrasion and facilitates egestion. In *X. laevis*, similar responses were previously recorded following exposure to polyester and PVC microplastics (Bacchetta et al. 2021, 2024).

Overall, the two species responded differently to PP microfiber exposure. *X. laevis* exhibited high ingestion and mild behavioural changes, while *O. virens* showed no ingestion but significant behavioural effects. These findings underscore the importance of using complementary models and behavioural endpoints in plastic toxicity studies. As emphasized by (Qualhato et al. 2023), incorporating a wider range of non-conventional species is crucial to better understand the risks posed by plastic pollution—especially in the wake of the COVID-19 pandemic.

## Conclusion

This study demonstrates that polypropylene microfibers derived from surgical mask degradation do not induce acute toxicity in *X. laevis* and *O. virens* in terms of mortality or major developmental and cell/tissue impairment. However, both species exhibited sublethal effects, ranging from gut accumulation in *X. laevis* to behavioral changes in both species: lowest observed effect concentrations (LOECs) were identified as 0.1 µg mL^-1^ and 10 µg mL^-1^ for *X. laevis* and *O. virens*, respectively. These results emphasize the species-specific response of differrent organisms to micro/nano plastic pollution and the environmental relevance of these concentration levels. In fact, LOEC concentrations fall within or near environmentally relevant levels, considering hot-spot contamination conditions (Saliu et al. 2021; found a mean release of 8 mg of microfibers from aged face masks, shaken in 1 L of seawater for 24 h). Future research could be addressed to: (i) elucidate possible mechanistic interpretation of the observed effects through the analyses of critical physiological indicators (e.g., oxidative stress, inflammatory responses); (ii) understand the actual source of toxicity of face mask microfibers i.e. the toxicity of micro/nanoplastics themselves the release toxicity of additives released within organisms, or the toxicity of additives leached in the aquatic environment. The complexity of micro/nanoplastic toxicity still require new research approaches and new efforts in order to better elucidate long-term ecological and physiological impacts. The Limitations of this study mainly concern the restricted exposure time in relation to the lifespan of the considered organisms and the specific life stage of the two organisms during exposure. Experimental constraints cannot be avoided, and they must be considered in interpreting the results. However, our results support the importance of multi-species and multi-endpoint studies to improve current knowledge on micro/nanoplastic toxicity and to better inform regulatory frameworks on this issue.

## Supplementary Information

Below is the link to the electronic supplementary material.

Supplementary Material 1

## Data Availability

Data will be made available upon request to the corresponding author.
