# Supplementary material for: Effects of polypropylene micro- and nanoplastics from surgical masks in amphibian and echinoderm models
Source: Ecotoxicology. 2026 Apr 2;35(5):93. doi: 10.1007/s10646-026-03077-w (PMC13046599; doi:10.1007/s10646-026-03077-w)
Supplement: Supplementary file 1 — Supplementary Material 1 [file 10646_2026_3077_MOESM1_ESM.docx]

**Supporting Information for**

**Effects of Polypropylene Micro- and Nanoplastics from Surgical Masks in Amphibian and Echinoderm Models**

Renato Bacchetta ^a^, Clotilde Vacchelli ^a^, Arianna Pica ^a^, Francesco Saliu ^b^, Daniela Maggioni ^c^,

Francesco Bonasoro ^a^, Nadia Santo ^d^, Paolo Tremolada* ^a^, Michela Sugni ^a^

^a^ *Department of Environmental Science and Policy, Università degli Studi di Milano, Via Celoria, 26, 20133, Milan, Italy*

*^b^ Department of Earth and Environmental Sciences DISAT, Università degli Studi di Milano Bicocca, Piazza della Scienza, 1, 20126, Milan, Italy*

*^c^ Department of Chemistry, Università degli Studi di Milano, Via Golgi, 19, 20133, Milan, Italy*

*^d^ Unitech NOLIMITS, Imaging Facility, Università degli Studi di Milano, Via Golgi, 19, 20133, Milan, Italy*

* *Corresponding author: Paolo Tremolada,* [*paolo.tremolada@unimi.it*](mailto:paolo.tremolada@unimi.it)


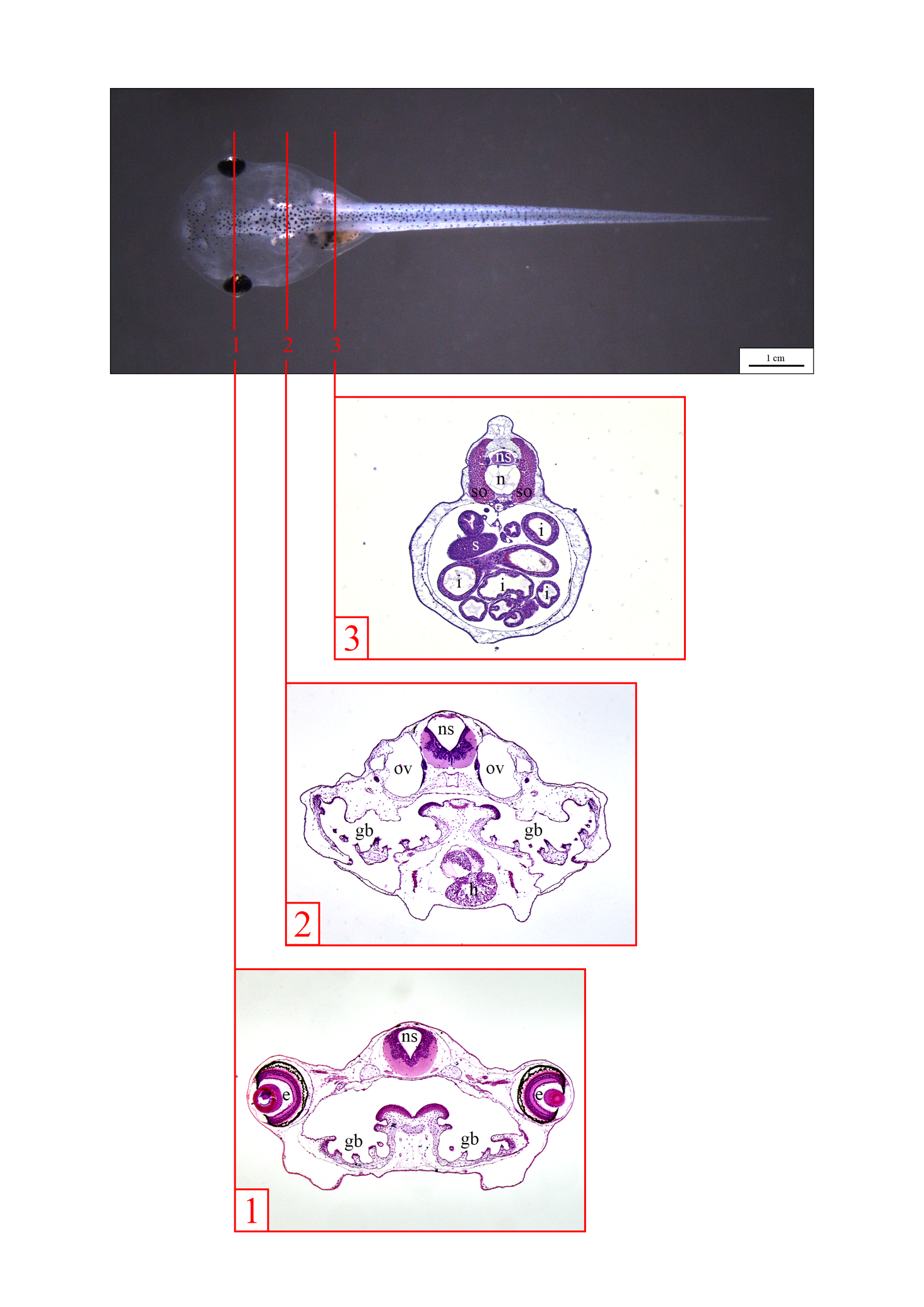


**Figure S1**. The three analysed portions of *X. laevis* larvae: (1) head (including eyes and gills), (2) ear vesicles and heart, (3) visceral area.

*Abbreviations*: ns = nervous system; e = eye; gb = gill basket; ov = otic vesicle; h = heart; n = notochord; so = somites; s = stomach; i = intestinal loop.


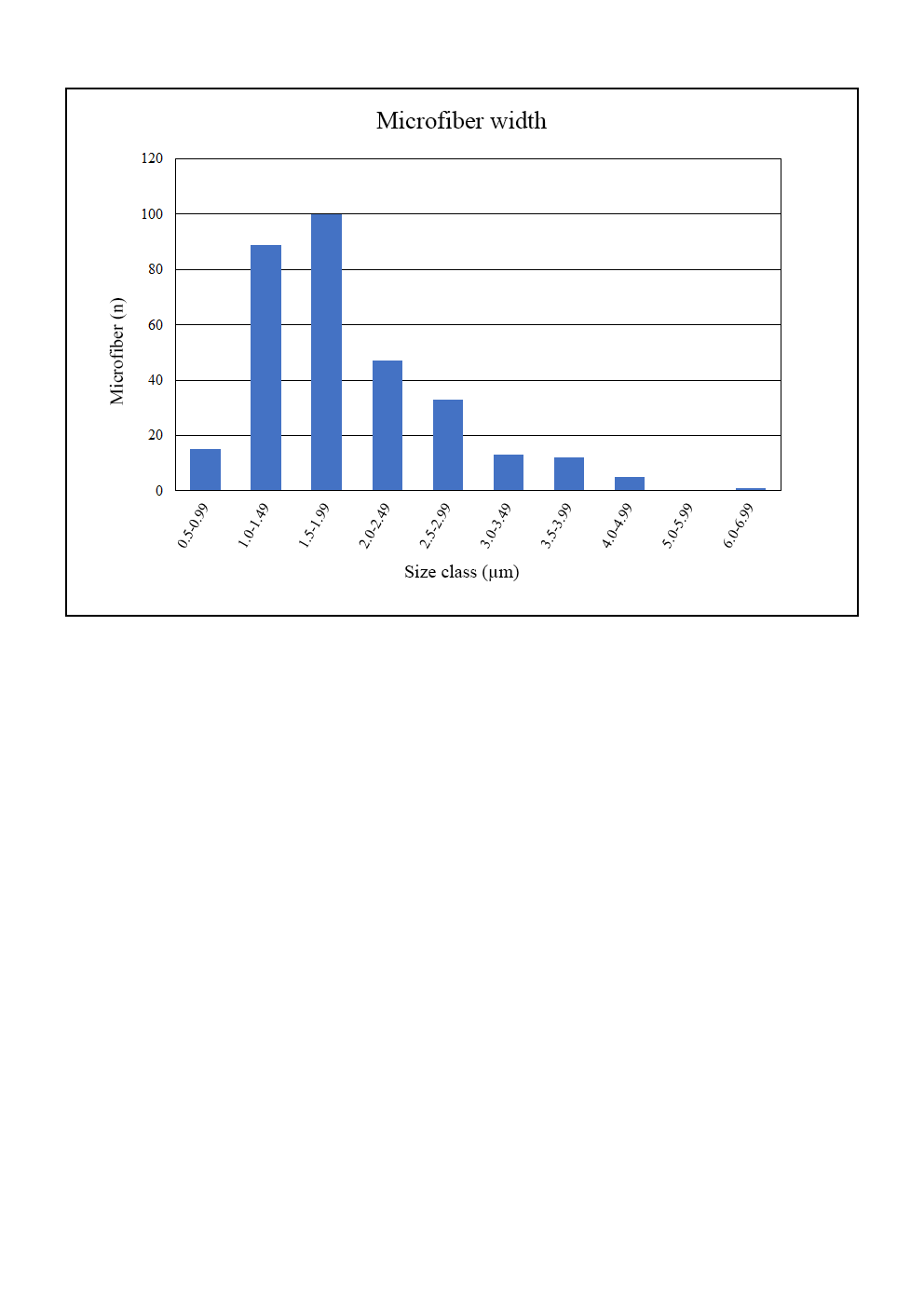


**Figure S2**. Histogram showing the width of PP microfibers in the different size classes.





**Figure S3.** SEM images of gill structure in *X. laevis* larvae. (A-B) Control larva at low and medium magnification. (C-D) High magnification of gills in two larvae exposed to the highest PP concentration (10 µg mL^-1^), showing isolated PP microfibers (white circles).

*Abbreviations*: gb = gill basket; g = gills

| **Table S1** - Embryotoxic effects of PP Face Mask microfibers on *X. laevis* larvae | | | | | | |  |
| --- | --- | --- | --- | --- | --- | --- | --- |
|  |  |  |  |  |  |  |  |
|  | Embryolethality | | |  | Teratogenicity | | |
|  | N. embryos | N. death | % mortality |  | N. living | N. malformed | % malformed |
| CTRL | 50 | 1 | 2 |  | 49 | 2 | 4.08 |
| 0.1 µg mL ^-1^ | 50 | 0 | 0 |  | 50 | 0 | 0 |
| 1 µg mL ^-1^ | 50 | 2 | 4 |  | 48 | 0 | 0 |
| 10 µg mL ^-1^ | 50 | 0 | 0 |  | 50 | 0 | 0 |

| **Table S2** - Mortality in *O. virens* exposure experiment | | | |
| --- | --- | --- | --- |
|  |  |  |  |
|  | n | n | n |
|  | specimens | deaths | living |
| CTRL | 20 | 2 | 18 |
| 0.1 µg mL ^-1^ | 20 | 7 | 13 |
| 1 µg mL ^-1^ | 20 | 4 | 16 |
| 10 µg mL ^-1^ | 20 | 4 | 16 |

**Table S3** - Larvae’s length (mm) of *Xenopus laevis* specimens at the end of the treatment in the 5 replicates for control (CTRL) and exposure batches (0.1, 1.0 and 10 μg mL^-1^).

| Replicates | *Xenopus laevis* larvae’s length (mm) | | | |
| --- | --- | --- | --- | --- |
|  | CTRL | 0.1 μg mL^-1^ | 1.0 μg mL^-1^ | 10 μg mL^-1^ |
| 1 | 10.778 | 10.899 | 10.763 | 10.634 |
|  | 10.253 | 10.576 | 10.623 | 10.805 |
|  | 10.325 | 10.509 | 10.956 | 10.553 |
|  | 10.32 | 10.684 | 11.12 | 10.222 |
|  | 10.666 | 10.806 | 11.067 | 10.614 |
|  | 9.936 | 10.483 | 10.516 | 10.76 |
|  | 10.562 | 10.737 | 11.16 | 10.757 |
|  | 10.555 | 10.889 | 10.896 | 10.322 |
|  | 10.784 | 10.571 | 10.926 | 10.524 |
|  | 10.738 | 11.092 | 10.531 | 10.442 |
| 2 | 10.771 | 10.571 | 10.671 | 10.718 |
|  | 10.865 | 10.552 | 10.953 | 10.671 |
|  | 10.294 | 10.586 | 10.679 | 10.38 |
|  | 10.865 | 10.609 | 10.617 | 10.829 |
|  | 10.393 | 10.319 | 10.825 | 10.583 |
|  | 10.551 | 10.933 | 10.536 | 10.837 |
|  | 10.102 | 9.727 | 10.896 | 10.642 |
|  | 10.259 | 10.783 | 10.94 | 10.979 |
|  | 10.883 | 10.634 | 10.557 | 10.644 |
|  |  | 11.067 | 10.511 | 10.932 |
| 3 | 10.558 | 10.583 | 10.968 | 10.847 |
|  | 10.975 | 11.062 | 10.429 | 10.788 |
|  | 10.396 | 10.482 | 10.507 | 10.866 |
|  | 10.006 | 10.93 | 10.585 | 10.764 |
|  | 10.91 | 10.531 | 11.011 | 10.775 |
|  | 10.69 | 10.66 | 10.516 | 10.948 |
|  | 10.4 | 10.526 | 10.62 | 11.114 |
|  | 10.647 | 10.811 | 10.464 | 10.748 |
|  | 9.665 | 10.585 | 10.793 | 10.775 |
|  | 10.568 | 10.848 | 10.63 | 10.935 |
| 4 | 11.054 | 10.734 | 10.278 | 10.819 |
|  | 10.734 | 10.813 | 10.725 | 10.247 |
|  | 10.346 | 10.779 | 10.575 | 11.058 |
|  | 10.648 | 10.893 | 10.535 | 10.617 |
|  | 10.481 | 10.594 | 11.126 | 10.731 |
|  | 10.622 | 10.764 | 10.511 | 10.754 |
|  | 10.657 | 10.807 | 10.859 | 10.869 |
|  | 10.468 | 10.747 | 10.755 | 10.559 |
|  | 10.669 | 10.299 | 11.134 | 10.686 |
|  | 10.549 | 10.545 | 10.52 | 10.53 |
| 5 | 11.052 | 10.764 | 10.685 | 10.849 |
|  | 9.63 | 10.734 | 10.343 | 10.991 |
|  | 10.871 | 10.208 | 10.793 | 11.219 |
|  | 10.624 | 10.546 | 10.564 | 11.257 |
|  | 10.98 | 11.02 | 10.882 | 10.619 |
|  | 10.782 | 10.495 | 10.557 | 10.534 |
|  | 10.74 | 10.951 | 10.751 | 10.706 |
|  | 10.754 | 11.087 | 11.049 | 10.684 |
|  | 10.764 | 11.002 |  | 10.69 |
|  | 9.744 | 11.031 |  | 10.184 |

**Table S4** – Behavioural parameters in *X. laevis* video tracking: total time spent (Total), time spent immobile (immob.) time spent swimming (swimm.); total distance travelled (Distance) and mean swimming speed (Speed) in the inner and outer part of the arena for the 5 replicates of control (CTRL) and exposure batches (0.1, 1.0 and 10 μg mL^-1^).

| Treat. | Repl. | Inner part | | | | |  | Outer part | | | | |
| --- | --- | --- | --- | --- | --- | --- | --- | --- | --- | --- | --- | --- |
|  |  | Total | Immob. | Swimm. | Distance | Speed |  | Total | Immob. | Swimm. | Distance | Speed |
|  |  | (s) | (S) | (S) | (mm) | (mm s^-1^) |  | (s) | (S) | (S) | (mm) | (mm s^-1^) |
| CTRL | 1.00 | 55.08 | 19.07 | 36.00 | 508.60 | 14.13 |  | 4.29 | 0.07 | 4.22 | 26.83 | 6.35 |
|  |  | 59.33 | 50.92 | 8.42 | 32.75 | 3.89 |  | 0.00 | 0.00 | 0.00 | 0.00 |  |
|  |  | 26.96 | 4.19 | 22.77 | 622.32 | 27.33 |  | 32.41 | 31.05 | 1.35 | 45.32 | 33.50 |
|  |  | 56.83 | 3.20 | 53.63 | 179.60 | 3.35 |  | 2.54 | 0.07 | 2.48 | 24.07 | 9.73 |
|  | 2.00 | 59.37 | 35.77 | 23.60 | 84.68 | 3.59 |  | 0.00 | 0.00 | 0.00 | 0.00 |  |
|  |  | 51.05 | 42.57 | 8.48 | 165.69 | 19.54 |  | 8.32 | 6.17 | 2.15 | 31.26 | 14.57 |
|  |  | 54.12 | 12.34 | 41.78 | 620.31 | 14.85 |  | 5.25 | 0.07 | 5.18 | 122.51 | 23.65 |
|  |  | 59.24 | 47.59 | 11.65 | 130.28 | 11.18 |  | 0.13 | 0.00 | 0.13 | 3.52 | 26.69 |
|  |  | 18.22 | 1.58 | 16.63 | 521.57 | 31.36 |  | 41.15 | 38.74 | 2.41 | 42.38 | 17.59 |
|  | 3.00 | 59.37 | 57.65 | 1.72 | 22.79 | 13.28 |  | 0.00 | 0.00 | 0.00 | 0.00 |  |
|  |  | 59.37 | 57.22 | 2.15 | 26.85 | 12.52 |  | 0.00 | 0.00 | 0.00 | 0.00 |  |
|  |  | 51.35 | 36.96 | 14.39 | 166.06 | 11.54 |  | 8.02 | 0.03 | 7.99 | 47.13 | 5.90 |
|  |  | 59.37 | 0.13 | 59.24 | 931.16 | 15.72 |  | 0.00 | 0.00 | 0.00 | 0.00 |  |
|  | 4.00 | 31.52 | 26.47 | 5.05 | 99.06 | 19.62 |  | 27.85 | 26.86 | 0.99 | 11.50 | 11.62 |
|  |  | 57.29 | 10.56 | 46.73 | 651.20 | 13.94 |  | 2.08 | 0.07 | 2.01 | 44.53 | 22.12 |
|  |  | 58.74 | 37.36 | 21.38 | 541.65 | 25.33 |  | 0.63 | 0.00 | 0.63 | 28.49 | 45.44 |
|  |  | 58.67 | 9.44 | 49.24 | 507.61 | 10.31 |  | 0.69 | 0.03 | 0.66 | 13.14 | 19.90 |
|  |  | 54.78 | 0.59 | 54.19 | 1140.81 | 21.05 |  | 4.59 | 0.17 | 4.42 | 113.51 | 25.67 |
|  | 5.00 | 57.62 | 27.13 | 30.49 | 405.37 | 13.29 |  | 1.75 | 0.07 | 1.68 | 46.84 | 27.83 |
|  |  | 56.69 | 0.20 | 56.50 | 1243.68 | 22.01 |  | 2.67 | 0.07 | 2.61 | 51.44 | 19.73 |
|  |  | 51.71 | 1.82 | 49.90 | 676.17 | 13.55 |  | 7.66 | 0.13 | 7.52 | 86.06 | 11.44 |
|  |  | 54.81 | 14.92 | 39.90 | 127.35 | 3.19 |  | 4.55 | 0.13 | 4.42 | 45.34 | 10.25 |
|  |  | 50.49 | 29.27 | 21.22 | 477.96 | 22.52 |  | 8.88 | 7.76 | 1.12 | 5.15 | 4.59 |
| 0.1 | 1.00 | 59.37 | 54.45 | 4.92 | 216.50 | 44.03 |  | 0.00 | 0.00 | 0.00 | 0.00 |  |
|  |  | 56.03 | 11.78 | 44.25 | 766.79 | 17.33 |  | 3.33 | 0.03 | 3.30 | 170.84 | 51.77 |
|  |  | 59.37 | 57.49 | 1.88 | 35.67 | 18.96 |  | 0.00 | 0.00 | 0.00 | 0.00 |  |
|  |  | 56.70 | 32.67 | 24.03 | 734.74 | 30.58 |  | 2.67 | 0.03 | 2.64 | 79.53 | 30.16 |
|  |  | 56.99 | 43.86 | 13.13 | 48.80 | 3.72 |  | 2.38 | 0.50 | 1.88 | 2.03 | 1.08 |
|  | 2.00 | 59.33 | 31.22 | 28.12 | 673.55 | 23.96 |  | 0.03 | 0.00 | 0.03 | 0.00 | 0.00 |
|  |  | 59.37 | 58.61 | 0.76 | 27.59 | 36.35 |  | 0.00 | 0.00 | 0.00 | 0.00 |  |
|  |  | 58.41 | 53.10 | 5.31 | 36.71 | 6.91 |  | 0.96 | 0.46 | 0.50 | 0.38 | 0.77 |
|  | 3.00 | 53.13 | 7.46 | 45.67 | 270.77 | 5.93 |  | 6.24 | 0.10 | 6.14 | 77.62 | 12.65 |
|  |  | 59.37 | 58.87 | 0.49 | 20.58 | 41.57 |  | 0.00 | 0.00 | 0.00 | 0.00 |  |
|  |  | 59.37 | 2.38 | 56.99 | 63.42 | 1.11 |  | 0.00 | 0.00 | 0.00 | 0.00 |  |
|  |  | 59.37 | 58.87 | 0.49 | 23.55 | 47.58 |  | 0.00 | 0.00 | 0.00 | 0.00 |  |
|  |  | 58.11 | 35.11 | 23.00 | 333.07 | 14.48 |  | 1.25 | 0.00 | 1.25 | 50.13 | 39.98 |
|  | 4.00 | 57.45 | 5.25 | 52.21 | 1265.00 | 24.23 |  | 1.91 | 0.00 | 1.91 | 52.24 | 27.29 |
|  |  | 58.54 | 39.34 | 19.21 | 427.64 | 22.27 |  | 0.83 | 0.00 | 0.83 | 38.90 | 47.15 |
|  |  | 59.37 | 58.87 | 0.49 | 24.46 | 49.41 |  | 0.00 | 0.00 | 0.00 | 0.00 |  |
|  | 5.00 | 59.37 | 56.86 | 2.51 | 33.40 | 13.32 |  | 0.00 | 0.00 | 0.00 | 0.00 |  |
|  |  | 59.37 | 51.55 | 7.82 | 166.78 | 21.33 |  | 0.00 | 0.00 | 0.00 | 0.00 |  |
|  |  | 59.37 | 58.87 | 0.49 | 9.44 | 19.07 |  | 0.00 | 0.00 | 0.00 | 0.00 |  |
|  |  | 59.24 | 0.07 | 59.17 | 856.28 | 14.47 |  | 0.13 | 0.00 | 0.13 | 2.01 | 15.20 |
|  |  | 55.44 | 21.58 | 33.86 | 283.36 | 8.37 |  | 3.93 | 0.03 | 3.89 | 37.16 | 9.54 |

**Table S4** – Continue

| Treat. | Repl. | Inner part | | | | |  | Outer part | | | | |
| --- | --- | --- | --- | --- | --- | --- | --- | --- | --- | --- | --- | --- |
|  |  | Total | Immob. | Swimm | Distance | Speed |  | Total | Immob. | Swimm | Distance | Speed |
|  |  | (s) | (S) | (S) | (mm) | (mm s^-1^) |  | (s) | (S) | (S) | (mm) | (mm s^-1^) |
| 1.0 | 1.00 | 54.85 | 0.17 | 54.68 | 997.00 | 18.23 |  | 4.52 | 0.10 | 4.42 | 55.59 | 12.57 |
|  |  | 58.08 | 0.07 | 58.01 | 754.28 | 13.00 |  | 1.09 | 0.03 | 1.06 | 20.51 | 19.43 |
|  |  | 44.48 | 41.05 | 3.43 | 25.85 | 7.53 |  | 14.88 | 4.82 | 10.07 | 28.34 | 2.82 |
|  |  | 45.41 | 28.41 | 17.00 | 38.76 | 2.28 |  | 13.96 | 12.77 | 1.19 | 8.48 | 7.14 |
|  |  | 55.31 | 34.91 | 20.39 | 342.90 | 16.81 |  | 4.06 | 0.07 | 3.99 | 59.45 | 14.89 |
|  | 2.00 | 59.37 | 58.87 | 0.49 | 23.72 | 47.91 |  | 0.00 | 0.00 | 0.00 | 0.00 |  |
|  |  | 57.59 | 0.13 | 57.45 | 335.09 | 5.83 |  | 1.78 | 0.03 | 1.75 | 12.13 | 6.93 |
|  |  | 59.37 | 58.87 | 0.49 | 29.45 | 59.50 |  | 0.00 | 0.00 | 0.00 | 0.00 |  |
|  |  | 57.45 | 3.83 | 53.63 | 632.74 | 11.80 |  | 1.91 | 0.07 | 1.85 | 45.87 | 24.82 |
|  | 3.00 | 57.62 | 0.10 | 57.52 | 848.71 | 14.76 |  | 1.75 | 0.03 | 1.72 | 53.79 | 31.35 |
|  |  | 59.37 | 0.03 | 59.33 | 1084.46 | 18.28 |  | 0.00 | 0.00 | 0.00 | 0.00 |  |
|  |  | 59.37 | 27.69 | 31.68 | 541.43 | 17.09 |  | 0.00 | 0.00 | 0.00 | 0.00 |  |
|  |  | 56.66 | 31.28 | 25.38 | 289.06 | 11.39 |  | 2.71 | 0.07 | 2.64 | 20.08 | 7.60 |
|  |  | 59.37 | 55.80 | 3.56 | 162.55 | 45.61 |  | 0.00 | 0.00 | 0.00 | 0.00 |  |
|  | 4.00 | 59.37 | 47.95 | 11.42 | 79.73 | 6.98 |  | 0.00 | 0.00 | 0.00 | 0.00 |  |
|  |  | 59.37 | 48.31 | 11.06 | 28.05 | 2.54 |  | 0.00 | 0.00 | 0.00 | 0.00 |  |
|  |  | 59.37 | 58.87 | 0.49 | 22.66 | 45.78 |  | 0.00 | 0.00 | 0.00 | 0.00 |  |
|  |  | 56.43 | 55.94 | 0.49 | 14.35 | 28.99 |  | 2.94 | 2.44 | 0.50 | 1.20 | 2.42 |
|  |  | 58.34 | 33.07 | 25.28 | 135.85 | 5.37 |  | 1.02 | 0.03 | 0.99 | 11.08 | 11.19 |
|  | 5.00 | 49.40 | 3.93 | 45.47 | 196.58 | 4.32 |  | 9.97 | 3.86 | 6.11 | 35.33 | 5.79 |
|  |  | 59.37 | 58.87 | 0.49 | 22.16 | 44.77 |  | 0.00 | 0.00 | 0.00 | 0.00 |  |
|  |  | 59.27 | 24.45 | 34.82 | 419.89 | 12.06 |  | 0.10 | 0.00 | 0.10 | 2.65 | 26.78 |
| 10 | 1.00 | 59.37 | 58.84 | 0.53 | 22.61 | 42.82 |  | 0.00 | 0.00 | 0.00 | 0.00 |  |
|  |  | 37.59 | 22.37 | 15.21 | 178.96 | 11.76 |  | 21.78 | 18.94 | 2.84 | 48.28 | 17.01 |
|  |  | 55.84 | 35.41 | 20.43 | 106.03 | 5.19 |  | 3.53 | 0.10 | 3.43 | 47.64 | 13.88 |
|  |  | 16.60 | 15.97 | 0.63 | 8.24 | 13.14 |  | 42.77 | 42.04 | 0.73 | 14.95 | 20.59 |
|  | 2.00 | 59.37 | 58.87 | 0.49 | 23.05 | 46.56 |  | 0.00 | 0.00 | 0.00 | 0.00 |  |
|  |  | 34.68 | 17.23 | 17.46 | 298.56 | 17.10 |  | 24.68 | 12.18 | 12.51 | 75.98 | 6.08 |
|  |  | 59.37 | 40.52 | 18.84 | 41.03 | 2.18 |  | 0.00 | 0.00 | 0.00 | 0.00 |  |
|  |  | 57.59 | 36.83 | 20.76 | 44.69 | 2.15 |  | 1.78 | 0.03 | 1.75 | 25.38 | 14.51 |
|  | 3.00 | 59.17 | 29.87 | 29.30 | 591.94 | 20.20 |  | 0.20 | 0.00 | 0.20 | 3.07 | 15.48 |
|  |  | 55.28 | 18.91 | 36.37 | 950.93 | 26.15 |  | 4.09 | 0.07 | 4.03 | 60.08 | 14.92 |
|  |  | 57.06 | 10.46 | 46.60 | 914.99 | 19.64 |  | 2.31 | 0.10 | 2.21 | 53.07 | 24.00 |
|  |  | 59.37 | 8.48 | 50.89 | 120.75 | 2.37 |  | 0.00 | 0.00 | 0.00 | 0.00 |  |
|  |  | 0.00 | 0.00 | 0.00 | 0.00 |  |  | 59.37 | 58.87 | 0.49 | 17.82 | 36.00 |
|  | 4.00 | 59.37 | 45.87 | 13.50 | 43.85 | 3.25 |  | 0.00 | 0.00 | 0.00 | 0.00 |  |
|  |  | 58.48 | 50.49 | 7.99 | 87.79 | 10.99 |  | 0.89 | 0.03 | 0.86 | 15.64 | 18.22 |
|  |  | 15.44 | 6.14 | 9.31 | 273.78 | 29.42 |  | 43.92 | 40.95 | 2.97 | 62.19 | 20.94 |
|  | 5.00 | 59.37 | 58.61 | 0.76 | 34.70 | 45.72 |  | 0.00 | 0.00 | 0.00 | 0.00 |  |
|  |  | 38.21 | 37.69 | 0.53 | 12.53 | 23.73 |  | 21.15 | 19.80 | 1.35 | 6.41 | 4.74 |
|  |  | 59.37 | 46.56 | 12.80 | 45.58 | 3.56 |  | 0.00 | 0.00 | 0.00 | 0.00 |  |
|  |  | 58.71 | 0.56 | 58.15 | 663.13 | 11.40 |  | 0.66 | 0.03 | 0.63 | 7.25 | 11.56 |

**Table S5** – Righting time (s) of *Ophiactis virens* specimens at the end of the treatment in the 2 replicates for control (CTRL) and exposure batches (0.1, 1.0 and 10 μg mL^-1^).

| Replicates | Righting time (s) | | | |
| --- | --- | --- | --- | --- |
|  | CTRL | 0.1 μg mL^-1^ | 1.0 μg mL^-1^ | 10 μg mL^-1^ |
| 1 | 16.54 | 37.78 | 7.70 | 6.10 |
|  | 7.55 | 6.99 | 9.06 | 22.64 |
|  | 9.82 | 17.12 | 11.13 | 13.49 |
|  | 5.00 | 6.64 | 7.01 | 37.43 |
|  | 13.68 | 3.68 | 5.59 | 19.94 |
|  | 25.09 | 15.96 | 12.76 | 25.65 |
|  | 7.69 | 13.89 | 8.61 | 8.22 |
|  | 4.71 |  | 7.99 | 11.86 |
|  | 6.38 |  |  | 7.04 |
| 2 | 3.56 | 8.37 | 6.85 | 16.01 |
|  | 7.48 | 10.14 | 4.73 | 21.18 |
|  | 10.17 | 10.80 | 18.06 | 7.41 |
|  | 9.20 | 4.96 | 17.40 | 9.38 |
|  | 4.94 | 21.31 | 7.67 | 8.45 |
|  | 8.85 | 8.02 | 4.41 | 12.71 |
|  | 3.84 |  | 5.92 | 12.72 |
|  | 6.00 |  | 11.24 |  |
|  | 9.34 |  |  |  |
